# Intraepithelial lymphocytes subsets in different forms of celiac disease

**DOI:** 10.1007/s13317-016-0085-y

**Published:** 2016-09-23

**Authors:** M. Sánchez-Castañon, B. G. Castro, M. Toca, C. Santacruz, M. Arias-Loste, P. Iruzubieta, J. Crespo, Marcos López-Hoyos

**Affiliations:** 1 Immunology Section, Hospital Universitario Marqués de Valdecilla-IDIVAL, 39008 Santander, Spain; 2 Gastroenterology Service, Hospital Universitario Marqués de Valdecilla-IDIVAL, Santander, Spain

**Keywords:** Intraepithelial lymphocytes, Flow cytometry, Active celiac disease, Silent celiac disease

## Abstract

**Aim:**

The enumeration of intraepithelial lymphocytes subsets (total, γδ, and CD3^–^ IELs) by flow cytometry (FCM), named as IEL lymphogram, constitutes a useful tool for celiac disease (CD) diagnosis. The aim of this study was to quantify IELs by FCM and their diagnostic value to differentiate active, silent and potential CD.

**Methods:**

Prospective study of 60 active and 20 silent CD patients, and 161 controls in which duodenal biopsy and IEL quantification by FCM was performed.

**Results:**

Active and silent CD patients had significant higher levels of both total and γδ IELs than absent CD patients (*P* < 0.0001 and *P* < 0.0001, *P* = 0.012 and *P* < 0.011; respectively). Active and silent CD patients had significant lower levels of CD3^–^ IELs than absent CD patients (*P* < 0.047 and *P* < 0.009, respectively). Moreover, they were lower in silent than in active CD patients (*P* = 0.002). Changes of IELs subsets were more marked in children than adults active CD. The optimal IEL lymphogram cut off values for active CD diagnosis were: ≥10, ≥15 and ≤9 %, and with better performance characteristics for silent CD: ≥ 11, ≥10 and ≤5 %.

**Conclusion:**

The evaluation of IELs subsets by FCM is useful to confirm diagnosis of active and silent CD.

## Introduction

Celiac disease (CD) is an autoimmune enteropathy induced by gluten in genetically predisposed patients and characterized by intestinal inflammation with lymphocytic intraepithelial infiltration and villous atrophy [1].

Intraepithelial lymphocytes (IELs) represent an abundant and heterogeneous population of antigen-experienced T cells that reside in the intestinal epithelium. They are composed of T cells bearing the αβ (αβ IELs >90 %) or the γδ T cell receptor (γδ IELs <10 %). Additionally, the epithelium of healthy intestinal mucosa contains a significant proportion of CD3^−^ CD103^+^ IELs (CD3^–^ IELs) [2].

It has been reported that IELs are increased in the duodenal/jejunum mucosa of untreated CD patients and would represent the earliest feature even seen when the villous architecture is preserved. Consequently, they alone can constitute the first sign of latent or potential forms of CD [3–6]. However, it can also be found in other autoimmune disorders, viral infections and diverse gastrointestinal diseases, such as small intestinal bacterial overgrowth (SIBO), tropical sprue, microscopic colitis, inflammatory bowel disease, irritable bowel syndrome (IBS) and allergic enteritis. Therefore, increased IELs are considered a hallmark of CD, although not disease-specific [2, 7].

Besides, increased γδ IELs can be found in the intestinal epithelium of both treated and untreated CD patients, and they are considered highly sensitive and specific for all the forms of CD. However, they are not pathognomonic, because they are also found in cow milk-sensitive enteropathy, food allergy, Crohn’s disease, post-enteritis syndrome, cryptosporidiasis and IgA-deficiency [8–11]. On the other hand, CD3^−^ IELs are present in healthy duodenal mucosa and disappear in active CD [12–15]. Therefore, quantification of both γδ and CD3^–^ IELs confers specificity to the finding of increased IELs observed in CD patients [12, 16]. Thus, the enumeration of IELs and the immunophenotyping of IELs subsets (total, γδ, and CD3^−^ IELs) by flow cytometry (FCM), named IEL lymphogram, constitute useful diagnostic tools for CD diagnosis but also to evaluate the activity of the disease and to identify atypical (latent and potential CD) and even asymptomatic forms (silent CD) [6].

The aim of this prospective study was to evaluate the diagnostic performance of quantifying the IELs subsets (total, γδ, and CD3^−^ IELs) by FCM in patients with active and silent CD, and to study differences between children and adults with active CD from a referral university hospital.

## Methods

This prospective study involved 231 patients with clinical suspicion for CD who underwent a duodenal biopsy and serologic studies for CD at the Pediatrics and the Gastroenterology outpatient clinics at the “Hospital Universitario Marques de Valdecilla” between June 2008 and April 2012. The work has been carried out in accordance with the local Ethics Committee. Clinical and histological data were collected from information contained in medical records.

We subdivided patients according to the type of CD into the following groups: active CD (*n* = 60) and silent CD (*n* = 20). We have also included 161 patients without CD (absent CD) in whom biopsy and serum tests were performed, as control group.

The diagnosis of CD was based on ESPGHAN and AGA criteria. The diagnosis of active CD required the presence of the classical symptoms of malabsorption, positive serology [anti-recombinant human tissue transglutaminase (TTG) and anti-gliadin (AGA, whole gliadin extract) IgA or IgG antibodies], as well as compatible histopathology and HLA-DQ2 and/or -DQ8. The diagnosis of silent CD was made in asymptomatic patients (with positive serology and villous atrophy).

**Fig. 1:**
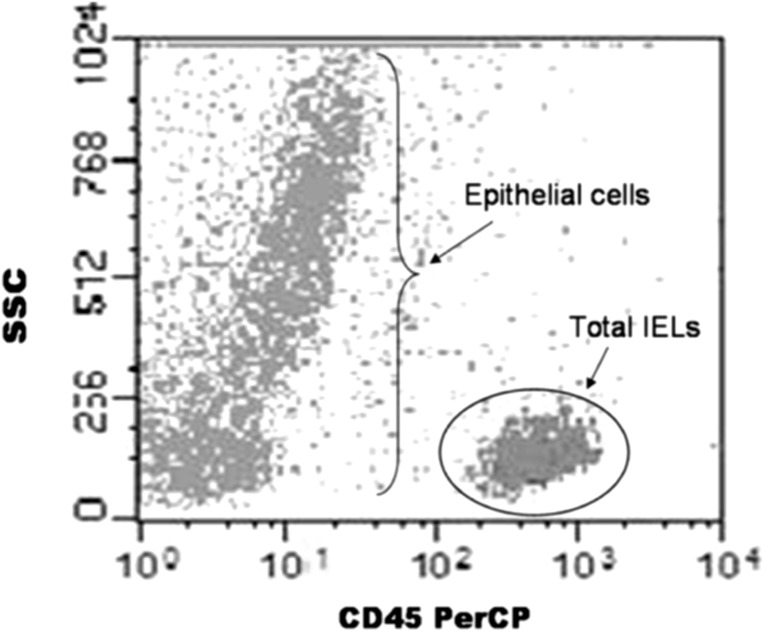
Selection of total IELs respect to the total cellularity of the duodenal epithelium, on the basis of their low 90° light scattering and CD45 expression

The combined study of total, γδ, and CD3^−^ IELs, termed IEL lymphogram, was performed by FCM in all patients. A single biopsy specimen was obtained for FCM analysis at the same time of diagnostic endoscopy for histological analysis. The sample was kept in physiologic saline solution, and immediately processed using a previously described protocol with minor modifications [17]. Briefly, IEL and epithelial cells were liberated from the mucosal specimens by mechanical disaggregation. The suspension of released cells was collected by centrifugation, washed and surface labelled with the appropriate fluorochrome-conjugated monoclonal antibodies (mAbs): CD45-PerCP, CD3-FITC, TcRγ δ-PE and CD103-PE (BD Biosciences, San Diego, CA). Data were acquired in a FACSCalibur (BD Biosciences) and analyzed with Cell Quest software (BD Biosciences). IEL cells were selected from the entire cell population of epithelial cells after gating on the basis of their low side scattering and CD45 expression (Fig. 1).

Duodenal biopsies were categorized according to Marsh classification as modified by Oberhuber et al. [14]. Lesions were further divided into two categories: mild, including increased intraepithelial lymphocytes, crypt hyperplasia, or mild villous atrophy (Marsh I, II, and IIIa); and severe, with marked villous atrophy, subtotal and total (Marsh IIIb and IIIc).

### Statistical analysis

Statistical analysis was carried out with the use of SPSS software (version 13, SPSS, Chicago, IL). All data were expressed as median ± interquartile range (IQR).

Differences in the percentage of the three IELs subsets (total, γδ and CD3^−^) among the different groups of CD and between children and adults active CD patients were calculated by the Mann–Withney *U* non-parametric test. This test was also used to calculate differences in the degree of histopathological lesions between the different groups of CD and between children and adults active CD.

The correlation among serum autoantibodies and total, γδ and CD3^−^ IELs frequencies in active and silent CD patients were calculated by the Spearman rho test. *P* values <0.05 were considered significant.

A receiver operator characteristic (ROC) curve analysis associated with the Youden index was carried out to determine the optimal cut off (CO) values for the combination of total, γδ and CD3^−^ IELs counts (IEL lymphogram) that could be used for the diagnosis of active, silent and potential CD. Diagnostic capacity was considered failed when area under the ROC curves (AUC) results were <0.5. The diagnostic value of IEL lymphogram was calculated as sensitivity, specificity, positive predictive value (PPV) and negative predictive value (NPV).

## Results

### Patient characteristics

The general characteristics of the patients included in the study are summarized in the Table 1. Among the 60 patients with active CD, 49 were children (82 %, female/male ratio = 1.6:1) and 11 were adults (18 %, female/male ratio = 4.5:1). However, from the patients with silent and absent CD, 3 out of 20 (15 %, female/male ratio = 2:1), and 11 out of 161 (7 %, female/male ratio = 1.6:1) were children, respectively.

**Table 1:** General characteristics of patients included in the study

	*n*	Age*	Sex (male/female)
Active celiac disease	60	9 (1–63)	21/39
Silent celiac disease	20	36 (12–62)	7/13
Absent celiac disease	161	42 (5–84)	58/103

* Data are expressed as mean years (range)

### IELs subsets counts in the different types of celiac disease

**Fig. 2:**
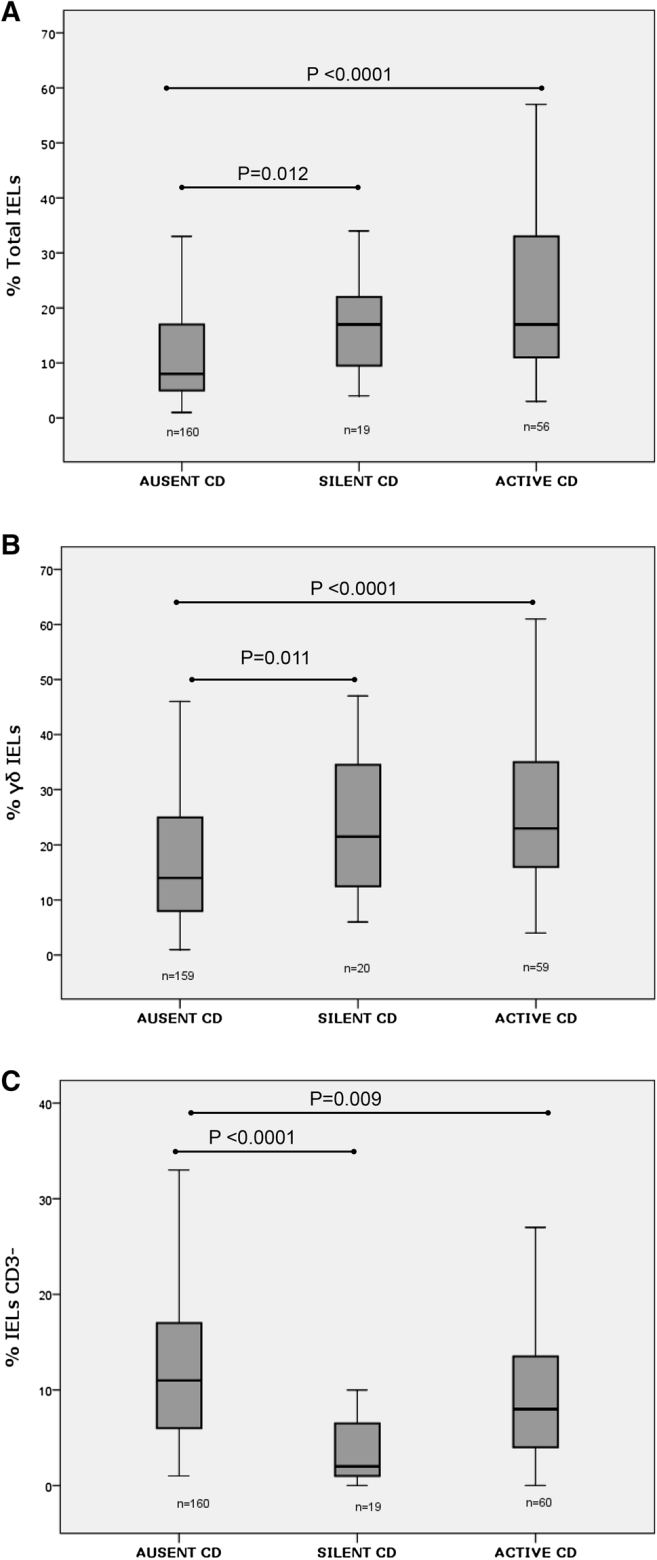
**a** Percentage of total IELs respect to the total cellularity of the duodenal epithelium in the study groups. *Box plots* with median, quartiles and 5 and 95 percentiles. *U* de Mann–Whitney test was used to detect significant differences (*P* < 0.05). **b** Percentage of γδ IELs relative to the total number of IELs in the study groups. *Box plots* show median, quartiles and 5 and 95 percentiles. *U* de Mann–Whitney test was used to detect significant differences (*P* < 0.05). **c** Percentage of CD3^–^ IELs relative to the total number of IELs in the study groups. *Box plots* show median, quartiles and 5 and 95 percentiles. *U* de Mann–Whitney test was used to detect significant differences (*P* < 0.05)

The median percentage of total IELs was significantly higher in both active CD and silent CD patients than in patients with absent CD. No significant differences were found among active and silent CD patients (Fig. 2a).

The median percentage of γδ IELs was also significantly higher in both active CD and silent CD patients than in patients with absent CD. No significant differences were found among active and silent CD patients (Fig. 2b).

The mean percentage of CD3^−^ IELs was significantly lower in both active CD and silent CD than in patients with absent CD. Moreover, it was significantly lower in silent CD than in active CD (Fig. 2c).

### IELs subsets counts in children and adult active celiac disease

Children had more total and γδ IELs (18 ± 22 and 27 ± 19 %, respectively) than adult active CD patients (11 ± 22 and 15.5 ± 14.5 %, respectively), although no significant differences were found among the groups (*P* = 0.176 and *P* = 0.088, respectively). On the contrary, the counts of CD3^−^ IELs were significantly lower in adult than in children active CD patients (4 ± 7 vs. 8 ± 13 %; *P* = 0.008).

### IELs and histopathology

The frequency of severe histopathology was significantly higher in patients with active CD (69 %) than in silent CD (35 %; *P* = 0.007). Severe active CD patients had higher levels of total IELs, γδ IELs and CD3^−^ IELs as compared with mild active CD patients. No significant differences were found in silent CD patients (Table 2).

**Table 2:** IELs populations and histopathology in active and silent CD

Active CD	Mild histopathology^a^ (*n* = 18)	Severe histopathology^b^ (*n* = 40)	*P* value*
% Total IELs	17.29 ± 14.28	25.95 ± 15.21	0.027*
% γδ IELs	21.22 ± 14.28	29.44 ± 15.08	0.031*
% CD3^–^ IELs	11.36 ± 19.57	11.62 ± 9.77	0.027*

Silent CD	Mild histopathology (*n* = 13)	Severe histopathology (*n* = 7)	*P* value*
% Total IELs	16.85 ± 7.29	14.00 ± 9.53	0.628
% γδ IELs	22.54 ± 12.93	31.57 ± 19.89	0.234
% CD3^–^ IELs	5.19 ± 6.35	2.61 ± 3.17	0.562

Data are expressed as mean ± standard deviation

*CD* celiac disease

* Significant differences (*P* < 0.05)

^a^Mild histopathology: Marsh I, II, and IIIa

^b^Severe histopathology: Marsh grade IIIb and IIIc

### IELs and serological markers

The frequency of positive AGA IgA was similar in active (72 %) or silent CD patients (60 %), with no differences according to patients age (Tables 3, 4). On the other hand, the frequency of positive TTG IgA was higher in active CD patients than in silent CD patients (87 vs. 70 %, no significant differences).

**Table 3:** Serological markers in celiac disease

	AGA IgA+^a^	TTG IgA+^b^	AGA IgA+ and TTG IgA+
Active celiac disease	74 (40/54)	87 (47/54)	67 (36/54)
Silent celiac disease	60 (12/20)	70 (14/20)	55 (11/20)
Absent celiac disease	1 (2/156)	0 (0/156)	0 (0/156)

Data are expressed as percentage (number of positive cases/total number of cases)

*AGA IgA* anti-gliadin IgA antibodies, *TTG IgA* anti-recombinant human tissue transglutaminase IgA

^a^AGA IgA+ ≥ 20 UI/L

^b^TTG IgA+ ≥ 4 UI/L

**Table 4:** Serological markers in children and adult celiac disease

	AGA IgA+^a^	TTG IgA+^b^	AGA IgA+ and TTG IgA+
Children (<18 years)			
Active celiac disease	77 (33/43)	91 (39/43)	70 (30/43)
Silent celiac disease	67 (2/3)	100 (3/3)	67 (2/3)
Absent celiac disease	0 (0/13)	0 (0/13)	0 (0/13)
Adult (≥18 years)			
Active celiac disease	64 (7/11)	73 (8/11)	55 (6/11)
Silent celiac disease	59 (10/17)	65 (11/17)	53 (9/17)
Absent celiac disease	0 (0/143)	0 (0/143)	0 (0/143)

Data are expressed as percentage (number of positive cases/total number of cases)

*AGA IgA* anti-gliadin IgA antibodies, *TTG IgA* anti-recombinant human tissue transglutaminase IgA

^a^AGA IgA+ ≥ 20 UI/L

^b^TTG IgA+ ≥ 4 UI/L

As serum levels of TTG IgA increased, a linear tendency toward higher γδ IELs counts in active CD patients (Spearman *ρ* = 0.422, *P* = 0.002) and more severe histopathological lesions (Spearman *ρ* = 0.393, *P* = 0.003) were observed. On the contrary, no correlation was found in silent CD patients.

### ROC curve for the IELs subsets in the diagnosis of active and silent CD

From the ROC curve analysis performed of IEL lymphogram in active CD diagnosis showed that the optimal CO values for the percentages of total, γδ and CD3^−^ IELs were: ≥10 % (sensitivity = 82 % and specificity = 59 %, AUC = 0.725), ≥15 % (sensitivity = 76 % and specificity = 53 %, AUC = 0.696) and ≤9 % (sensitivity = 67 % and specificity = 57 %, AUC = 0.614), respectively.

The optimal CO values for the percentage of total, γδ and CD3^−^ IELs for silent CD diagnosis were: ≥11 % (sensitivity = 74 % and specificity = 60 %, AUC = 0.673), ≥10 % (sensitivity = 95 % and specificity = 33 %, AUC = 0.663) and ≤5 % (sensitivity = 74 % and specificity = 79 %, AUC = 0.836), respectively.

### ROC curve for the IELs subsets in the diagnosis of children and adult active CD

The ROC curve analysis performed on the IEL subsets for the children active CD diagnosis showed that the optimal CO values for the percentage of total, γδ and CD3^−^ IELs were: ≥10 % (sensitivity = 87 % and specificity = 62 %, AUC = 0.715), ≥15 % (sensitivity = 82 % and specificity = 77 %, AUC = 0.832) and ≤9 % (sensitivity = 63 % and specificity = 61 %, AUC = 0.612), respectively. Whereas for adult active CD patients were: ≥10 % (sensitivity = 60 % and specificity = 58 %, AUC = 0.587), ≥10 % (sensitivity = 90 % and specificity = 31 %, AUC = 0.547) and ≤4 % (sensitivity = 64 % and specificity = 80 %, AUC = 0.798), respectively.

### Diagnostic value of IEL lymphogram for active and silent CD diagnosis

Table 5 shows the sensitivity, specificity, PPV and NPV of the optimal IEL lymphogram (total, γδ, and CD3^−^ IELs counts) for active and silent CD diagnosis.

**Table 5:** Diagnostic value of optimal IEL lymphogram for active and silent CD

	Sensitivity (%)	Specificity (%)	PPV (%)	NPV (%)
Optimal IEL lymphogram in active CD (≥10, ≥15 and ≤9 %)	45	86	52	82
Optimal IEL lymphogram in silent CD (≥11, ≥10 and ≤5 %)	56	92	44	95

IEL lymphogram: total, γδ and CD3^–^ IELs

*IEL* intraepithelial lymphocytes, *CD* celiac disease, *PPV* positive predictive value, *NPV* negative predictive value

In this study, the optimal IEL lymphogram for silent CD diagnosis (total IELs ≥11 %, γδ IELs ≥10 % and CD3^−^ IELs ≤5 %) had higher sensitivity, specificity and NPV than the IEL lymphogram for active CD diagnosis (total IELs ≥10 %, γδ IELs ≥15 % and CD3^−^ IELs ≤9 %). However, the PPV of IEL lymphogram was higher for active CD than for silent CD diagnosis (52 vs. 44 %).

## Discussion

CD is an immune-mediated enteropathy caused by an abnormal immune response to dietary gluten proteins, characterized by altered IELs pattern.

Camarero et al. [13] have developed a FCM technique for the evaluation of IELs subsets in intestinal biopsies of CD patients. Three main alterations have been described in CD patients. First, total IELs are increased during the active phases of the disease [3–6, 18]. Second, which could be considered practically pathognomonic, there is a permanent increase in γδ IELs [19, 20], observed in every form of the disease [8, 13, 21], and it could constitute the first sign of latent or potential forms of CD [9]. Finally, there is a pronounced and constant decrease in CD3^−^ IELs, which become virtually undetectable in active CD [12, 23–25]. Since those changes in IEL subsets are very characteristic of CD, the analysis by FCM of the 3 parameters (total, γδ and CD3^−^ IELs), and termed “IEL lymphogram” has been described to be of high specificity and sensitivity in the diagnosis of CD, even of atypical forms [6].

In this study we have evaluated the profile of IELs in a long cohort of patients with active and silent CD. According to other authors, we have also found a significant increase of both total and γδ IELs and a significant decrease of CD3- IELs in active CD patients as compared to patients with absent CD [13, 16, 19, 22, 26]. Interestingly, we have observed similar alterations on IELs subsets in silent CD patients. Our results are in discordance with the study of Erias et al. [27] that reported the same IEL lymphograms in patients with active, latent and potential CD, and suggested that the IELs lymphogram may be particularly useful for the diagnosis of latent and potential CD. In the same way, Goldstein et al. [28] suggested that increased IELs could constitute, by themselves, the first sign of latent or potential forms of CD, even in the presence of preserved villi. Moreover, several authors reported that potential CD was characterized by an increased number of total and/or γδ IELs [9, 13, 21, 27]. From our data, the evaluation of the IELs subsets may be of help as diagnostic tool in active and silent CD, but we do not have conclusive data on potential CD.

Besides, we have compared the IELs subsets in children and adults active CD patients. Accordingly with the study of Calleja et al. [26], we have also found more pronounced changes on IELs subsets in children than in adult active CD patients, suggesting that the determination of IELs subsets may be particularly useful for the diagnosis of active CD in children.

Of note, we have observed that the increase of total and γδ IELs was higher in active CD patients with severe histopathology than in those with mild histopathology. Moreover, we have found that as serum levels of TTG IgA increase there is a linear tendency toward more severe histopathological lesions and higher γδ IELs counts. These data are in agreement with previous reports [29–32], which described that TTG IgA levels are correlated with the degree of histopathological lesions in CD patients. However, our results are in disagreement with previous studies [8, 13, 21, 26], which reported that the density of γδ IELs was not correlated with the degree of intestinal tissue damage and that there was an increase on the density of γδ IELs in all stages of CD, including latent and potential CD patients. These authors suggest that the evaluation of γδ IELs could be used as a diagnostic marker to identify early stage CD. Our data may reflect that in patients with more active disease, represented by more severe histopathology and higher levels of TTG IgA, there are more abnormalities in IELs subsets, mainly γδ IELs.

Finally, the optimal IEL lymphogram set in the present study for silent CD diagnosis (total IELs ≥11 %, γδ IELs ≥10 % and CD3^−^ IELs ≤5 %) had better diagnostic performance than that set for active CD diagnosis (total IELs ≥10 %, γδ IELs ≥15 % and CD3^−^ IELs ≤9 %).

In conclusion, our results indicate that the evaluation of IELs subsets by FCM could be useful to confirm diagnosis of active and silent CD.
